# Naturally-Acquired Influenza-Specific CD4^+^ T-Cell Proliferative Responses Are Impaired in HIV-Infected African Adults

**DOI:** 10.1371/journal.pone.0038628

**Published:** 2012-06-08

**Authors:** Kondwani C. Jambo, Enoch Sepako, Sarah J. Glennie, David Mzinza, Neil A. Williams, Stephen B. Gordon, Robert S. Heyderman

**Affiliations:** 1 Malawi-Liverpool-Wellcome Trust Clinical Research Programme, University of Malawi College of Medicine, Blantyre, Malawi; 2 Respiratory Infection Group, Liverpool School of Tropical Medicine, Liverpool, United Kingdom; 3 Cellular and Molecular Medicine, Medical Sciences Building, University of Bristol, Bristol, United Kingdom; Institut National de la Santé et de la Recherche Médicale U 872, France

## Abstract

**Background:**

Seasonal influenza has been associated with greater morbidity and mortality in AIDS patients. Highly-active antiretroviral therapy (HAART) has led to some reduction in influenza-related complications but the nature of naturally-acquired T-cell immunity to influenza virus in an African setting, and how this changes with immune reconstitution following HAART is unknown. We measured influenza-specific CD4^+^ T-cell immunity in unimmunized HIV-infected Malawian adults and then investigated immune reconstitution following HAART.

**Methods:**

Peripheral blood mononuclear cells were isolated from HIV-infected and HIV-uninfected Malawian adults. CFSE proliferation and CD154 expression flow cytometry-based assays were used to measure influenza-specific CD4^+^ T-cell immunity.

**Results:**

We found lower naturally-acquired proliferative influenza-specific CD4^+^ T-cell responses in AIDS patients that was also present in asymptomatic HIV-infected adults with relatively high CD4 counts (>350 cells/µl). Influenza-specific CD4^+^ T-cell immune reconstitution in HIV-infected patients on HAART for 12 months was poor despite a marked reduction in viral load and an increase in CD4 count. This poor immune reconstitution was characterised by a low influenza-specific proliferative CD4^+^ T-cell response and reduced proportions of CD154-expressing influenza-specific CD4^+^ T-cells in peripheral blood.

**Conclusion:**

Our data suggest that asymptomatic HIV-infected adults may also be at risk of influenza-related complications and that HAART alone may not circumvent this risk in AIDS patients. This study highlights the need to identify possible interventions early in HIV infection to reduce the risk of influenza and to intensify influenza surveillance in these susceptible African populations.

## Introduction

Influenza virus causes 0.5 million deaths each year and due to the emergence of new reassortants such as the 2009 H1N1 pandemic influenza A virus, are able to escape immune surveillance, thus posing a major threat to global health [1]. Most of the data on the burden of influenza infection and the characteristics of naturally-acquired immunity have emerged from Europe, North America and Southeast Asia [1]. Very little has been reported from sub-Saharan Africa (SSA), home to two-thirds of world's people living with HIV [2].

There is conflicting data regarding HIV as one of the risk factors for more severe disease and death in influenza patients [3], [4]. One of the possible reasons for the discrepancies in the studies has been the sample size, as most of the studies have been underpowered to address this question and have mostly been done in western countries. Recently, there has been a study from South Africa by Cohen *et al.* that has shown that there is an increased risk of death and longer mean duration of hospitalisation amongst HIV-infected persons hospitalised with influenza-confirmed illness than HIV-uninfected counterparts [Conference paper].

In industrialised countries, the introduction of highly-active antiretroviral therapy (HAART) has led to some reduction in influenza-associated complications among HIV-infected persons, which appears to be related to the increase in CD4 count [5]. In the context of a potential influenza epidemic in sub-Saharan Africa affecting large numbers of HIV infected individuals [6], the risk associated with HIV infection among those with relatively less immune suppression remains to be elucidated.

CD4^+^ T-cells are important in anti-influenza defence, they provide help to B-cells which leads to production of antibodies necessary to complete viral clearance [7]. They also promote the generation of memory influenza-specific CD8^+^ T-cells which help in clearing secondary influenza infections [8]. We have previously demonstrated strong naturally-acquired mucosal and systemic CD4^+^ T-cell immune memory to influenza antigens in healthy UK adults [9]. We have also shown that asymptomatic HIV-infected African adults have considerable signs of immune dysregulation with impaired influenza-specific CD4^+^ T-cell responses in the lung [10]. Here we further describe nature of systemic naturally-acquired CD4^+^ T-cell immunity in an African setting and assess how this changes with immune reconstitution with HAART. We observed a robust influenza-specific immunity in healthy African adults, which was lost in HIV-infected adults and was not restored even after 12months on HAART.

## Materials and Methods

### Ethical approval

The research described fulfil all institutional guidelines and was approved by the College of Medicine Bioethics Committee, Malawi (P.03/08/626 and P.01/09/717) and The Liverpool School of Tropical Medicine Research Ethics Committee (08.41 and 08.61.).

### Subjects

Two cohorts of adults with no recent history of severe respiratory diseases were recruited at the Queen Elizabeth Central Hospital (QECH), Blantyre, Malawi: a cohort of HIV-uninfected adults and asymptomatic HIV-infected individuals who were sampled once (cross-sectional cohort); and a cohort of HIV-infected adults about to start HAART (WHO stage 3/4) who were sampled just before starting HAART and sampled again 6months and 12months after starting HAART (longitudinal cohort). According to national guidelines, the commencement of HAART in Malawi is currently mostly based on clinical WHO staging rather than CD4 count. All participants gave written-informed-consent to HIV testing and blood collection. The exclusion criteria for the study were: use of immunosuppressive drugs, moderate or severe anaemia (HB<8 g/dl) and known or possible pregnancy. The influenza immunity data from HIV-uninfected adults and asymptomatic HIV-infected individuals has been reported in part previously [11].

### Assessment of influenza-specific CD4^+^ T-cell immunity

Peripheral blood mononuclear cells (PBMCs) were processed as previously described [12]. Influenza-specific CD4^+^ T-cell immune responses were determined using Carboxyfluorescein succinimidyl ester (CFSE) proliferation and CD154 (CD40L) expression assays. In all the assays, PBMCs were stimulated with 2008/2009 Inactivated Influenza vaccine (Split virion) BP (Sanofi Pasteur MSD, UK) (0.45–0.9 µg/ml) (the vaccine contains antigens equivalent to the following strains; A/Brisbane/59/2007 (H1N1)-like strain IVR-148 derived from A/Brisbane/59/2007, A/Brisbane/10/2007 (H3N3)-like strain NYMC X-175C derived from A/Uruguay/716/2007 and B/Florida/4/2006-like strain B/Florida/4/2006) as the test antigen or phytohemagglutinin (PHA) (0.5–4 µg/ml) as the positive control. Due to limitations in cell numbers, not all the assays were done on all the volunteers.

Proliferation of CD4^+^ T-cells was measured by flow cytometry after a CFSE dilution assay as previously described [11]. Briefly, PBMC were stained with CFSE, stimulated with antigens or left unstimulated, harvested after 7 days, surface-stained with anti-human CD3 Peridinin Chlorophyll Protein Complex (PercP) and CD4 allophycocyanin (APC) (All BD Biosciences), and acquired on a CyAn flow cytometer (Beckman Coulter, USA).

CD154 expression was measured using a co-culture method as described previously [11]. Briefly, PBMCs were co-cultured with anti-CD154-PE antibody (BD Biosciences), stimulated with antigens or left unstimulated for 16 hours, harvested, surface-stained with anti-CD3 Fluorescein isothiocyanate (FITC), anti-CD4-PerCP, anti-CD69-APC and anti-CD154-PE (BD Biosciences), and acquired on a flow cytometer. FACS analysis was done using FlowJo (Tree Star, USA).

### Statistical analyses

Statistical analyses and graphical presentation were done using GraphPad Prism 5 (GraphPad Software, USA). The data were analyzed using the Mann-Whitney *U* test (non-paired data) and Wilcoxon signed-ranked test (paired data). Results are given as medians with interquartile ranges (IQRs). Differences were considered statistically significant where p≤0.05.

## Results

### Demographic characteristics

In the cross sectional cohort, 31 HIV-uninfected adults (32% female; median age 33years(23–53)) and 31 asymptomatic HIV-infected patients (46% female; median age 31years(18–58)) were recruited. The median CD4 count in HIV-uninfected adults was 853cells/µl(444–1323) and in the asymptomatic HIV-infected patients was 252cells/µl(7–913). In the longitudinal HAART cohort, 10 HIV-uninfected adults (35% female; median age 31years(24–56)) and 16 HIV-infected patients about to commence HAART (56% female; median age 37years(25–55)) were enrolled. The median CD4 count in HIV-uninfected adults was 602cells/µl(531–763) and HIV-infected patients about to commence HAART was 160cells/µl(79–227) which increased to 249cells/µl(189–360) at 6months on HAART (p<0.0001), but did not significant increase at 12months (249cells/µl(189–360) vs. 274cells/µl(197–370); p>0.05). The viral load was either undetectable or below 5000 copies/ml in all participants at 6 months. None of the participants in either cohort had been vaccinated against influenza.

### Poor influenza-specific proliferative CD4^+^ T-cell responses in HIV-infected adults irrespective of CD4 count

**Figure 1:**
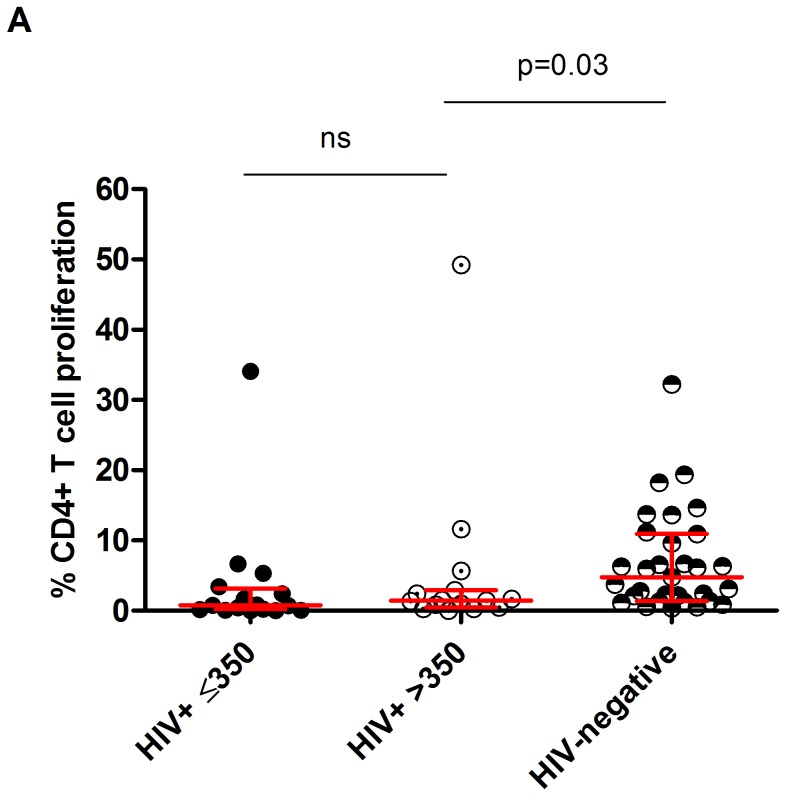
Impaired influenza virus antigen-specific CD4^+^ T-cell proliferative responses in peripheral blood of HIV-infected adults. (A) Peripheral blood mononuclear cells were stained with CFSE, stimulated with influenza vaccine for 7 days and proliferative responses were measured using flow cytometry. Black horizontal bars represent median and IQRs after background responses were subtracted from all antigen-specific CD4^+^ T-cell responses. Statistical significance was analysed by the Mann-Whitney U test (CD≤350, n = 16; CD>350, n = 15; HIV−, n = 31). The influenza immunity data from HIV-uninfected adults and asymptomatic HIV-infected individuals has been reported in part previously [11].

We evaluated the proliferative capacity of CD4^+^ T-cells in response to influenza antigens. We found that the influenza-specific proliferative CD4^+^ T-cell responses in the HIV-infected adults with CD4 count>350 were lower than those of HIV-uninfected individuals (1.44%[0.5–2.9] vs. 4.78%[1.4–10.9]; p = 0.03) (**Figure 1**). The influenza-specific proliferative CD4^+^ T-cell responses in HIV-infected patients were similar between those that had a CD4 count>350 and those with a CD4 count<350 (0.76%[0.1–3.2] vs. 1.44%[0.5–2.9]; p>0.05) (**Figure 1**). However, this impairment in CD4^+^ T-cell proliferative responses was not observed in response to polyclonal stimulation (PHA) confirming that there was no intrinsic defect in function of CD4 T-cells from HIV-infected individuals (**Figure S1**).

### Poor immune reconstitution of Influenza-specific CD4^+^ T-cell responses in HIV-infected patients following HAART

**Figure 2:**
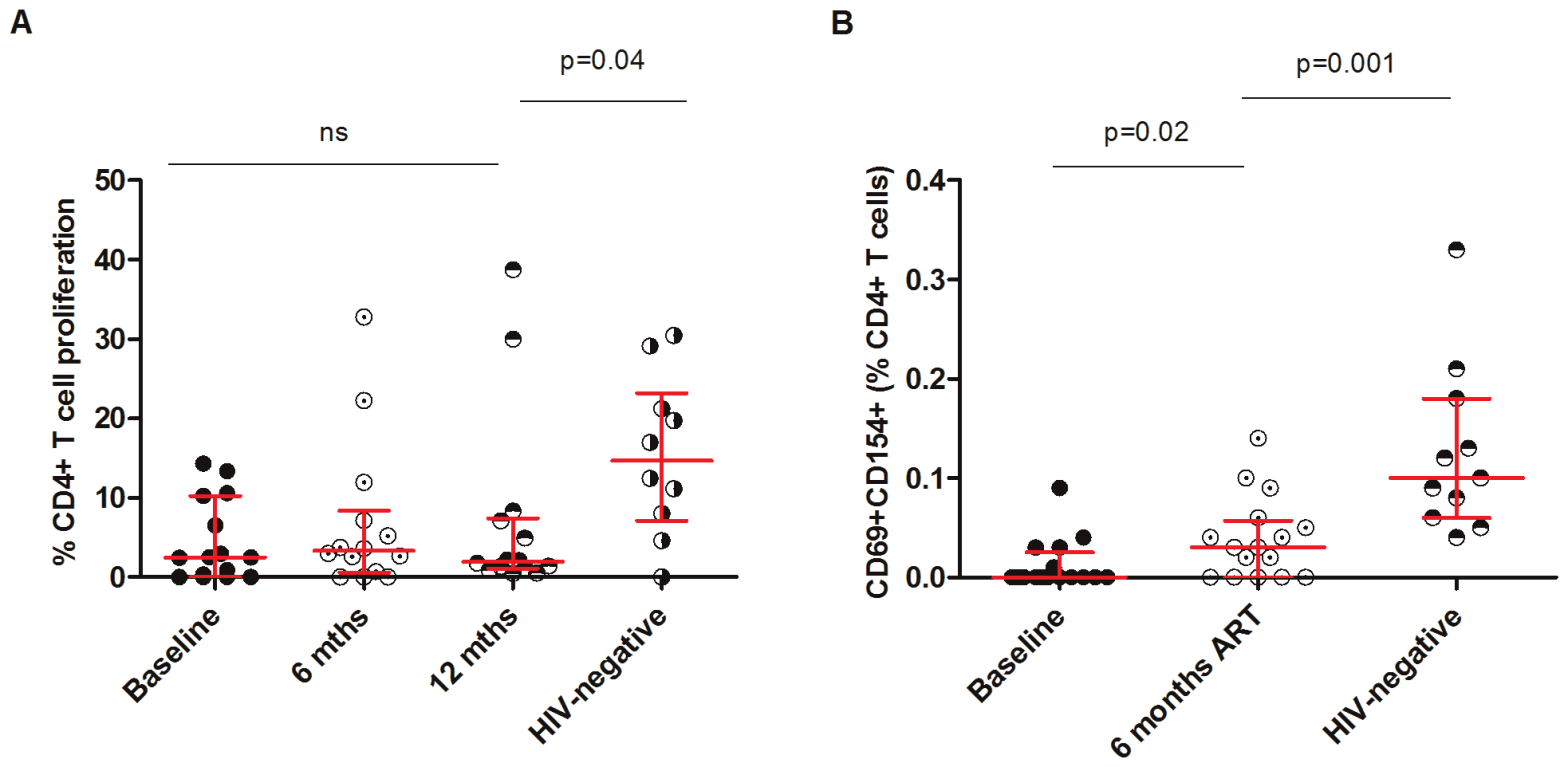
Impaired immune reconstitution in influenza antigen-specific CD4^+^ T-cell responses in peripheral blood of HIV-infected adults following HAART. (A) Peripheral blood mononuclear cells (PBMCs) were stained with CFSE, stimulated with influenza vaccine for 7 days and proliferative responses were measured using flow cytometry (Baseline, n = 15; 6months, n = 14; 12months, n = 14; HIV−, n = 10) (B) PBMCs were stimulated with influenza vaccine and the expression of CD154 on activated CD4^+^ T cells was measured using flow cytometry. Black horizontal bars represent median and IQRs after background responses were subtracted from all antigen-specific CD4^+^ T cell responses (Baseline, n = 16; 6months, n = 16; 12months, n = 11; HIV−, n = 11). Statistical significance was analysed by the Mann-Whitney U test.

Following the observations that influenza-specific proliferative responses were impaired in HIV-infected patients, we investigated whether HAART reversed this immune defect. The proliferative influenza-specific responses at 12months following HAART were lower than HIV-uninfected individuals (1.9%[1.1–7.4] vs. 14.7%[7.2–23.2]; p = 0.04). The proliferative influenza-specific responses in HIV-infected patients at 6months following HAART were similar with those detected at baseline (3.3%[0.6–8.3] vs. 2.5%[0.0–10.2]; p>0.05), and did not differ with those detected at 12months (3.3%[0.6–8.3] vs. 1.9%[1.1–7.4]; p>0.05) (**Figure 2A**). There was no association between CD4 nadir or viral load and antigenic proliferative response at baseline, 6months or 12months (p>0.05 for all) (data not shown).

We also measured CD154 expression, pre- and post-HAART. CD154 is an important molecule expressed on activated antigen-specific CD4^+^ T-cells and is involved in activation of antigen-presenting cells, priming of CD4^+^ T-cells, B-cell proliferation and immunoglobulin synthesis. Due to its various functions, CD154 it likely to play an important role in defence against influenza infection. Induction of CD154 in response to microbial pathogens is impaired in CD4^+^ T cells from HIV-infected patients and this has been associated with increased susceptibility to these pathogens [13]. CD154 can also be used as marker to detect the proportion antigen-specific CD4^+^ T-cell s, as it is expressed on recently activated antigen-specific cells following stimulation with cognate antigens [14]. We found some degree of immune reconstitution in the frequency of activated influenza-specific CD4^+^ T-cells expressing CD154 in HIV-infected patients following 6 months on HAART (0.0%[0.0–0.03] vs. 0.03%[0.0–0.06]; p = 0.03), however, the frequency was still lower than HIV-uninfected individuals (0.03%[0.0–0.06] vs. 0.1%[0.06–0.2]; p = 0.001) (**Figure 2B**).

## Discussion

Pandemic influenza A has had devastating effects amongst young fit adults [15], however, typically the very young, the very old, pregnant women and immunocompromised individuals have had the worst outcome [16]. In many industrialised countries these at-risk groups are targeted for influenza vaccination. We have demonstrated widespread influenza-specific CD4^+^ T-cell immunity in a healthy African adult population, which is impaired in HIV-infected individuals even after immune reconstitution with HAART for 12months.

Outside South Africa [17], there is limited information on the prevalence of influenza virus infections. The observation that there is widespread robust influenza-specific immunity in healthy Malawian adults and indeed children (unpublished data) in the absence of vaccination, suggests that there is considerable exposure to influenza virus in this population. These African adults had similar immunity to their counterparts in industrialised countries where prevalence of influenza infections are high [18]. CD4^+^ T-cells generated from priming with seasonal influenza viruses and vaccines cross-react with antigens derived from pandemic H1N1 virus [19]. Although the influenza-specific CD4^+^ T-cells generated from seasonal influenza strains might provide some degree of cross-protection to pandemic strains, the uncertainty of the extent and geographical spread of influenza infection in this population poses a major challenge to preparedness to a potential influenza pandemic.

In contrast to the influenza-specific CD4^+^ T-cell immunity observed in the HIV-uninfected group, we observed impaired influenza-specific CD4^+^ T-cell proliferative responses in HIV-infected individuals, a measure of central memory [20]. We have previously shown that HIV-infected adults had no depreciation in peripheral blood influenza-specific cytokine-secreting effector memory CD4^+^ T-cells. This variance in results from the two different assay is consistent with the evidence that there is preferential depletion of central memory versus effector memory T cells in HIV-infected adults [11], [21]. We speculate, therefore, that the influenza-specific proliferative CD4+ T-cell responses are impaired earlier than effector responses during HIV infection.

Interestingly, we found that influenza-specific CD4^+^ T-cell function was already lost in individuals with relatively high CD4 count and no association was found between CD4 count and influenza-specific proliferative responses, showing that defects in influenza-specific CD4^+^ T-cell proliferative response happens early in HIV-infection before a global depletion of CD4^+^ T-cells. Given the likely effects on antigen presentation and antibody mediated immunity [22], [23], the implications of poor influenza immunity in HIV-infected individuals goes beyond the potential threat of an H1N1 epidemic [1]. Epidemiological evidence and mouse infection models suggest that influenza predisposes to a range of invasive bacterial infections, including pneumococcal pneumonia [24]. Impaired influenza-specific immunity in early HIV infection in an area of intense pneumococcal exposure may contribute to increased incidences of pneumococcal disease. Most likely any vaccine intervention will need to be introduced early in the course of HIV infection in order to circumvent the risk of influenza-related bacterial complications.

Although HAART has helped in reducing morbidity and mortality due to influenza-related complications in some populations [5], influenza virus is still among the leading causative agents of febrile respiratory illnesses among HIV-infected outpatients on HAART in industrialised countries [25]. We have demonstrated an impaired influenza-specific proliferative CD4^+^ T-cell response and modest reconstitution in CD154 expression in individuals on HAART for 6 months despite an increase in CD4 count and a reduction in HIV viral load. This might be due to commencement of HAART with a low CD4 count nadir, which has been associated with partial immune reconstitution and poor outcome [26]. It might also be that HAART alone is not sufficient to reconstitute influenza-specific responses. This evidence of poor immune influenza-specific reconstitution, suggests that HIV-infected patients on HAART may be at risk to influenza-related complications. Ensuring early commencement of HAART and targeted vaccination strategies early on in the course of HIV infection may help reduce the risk of influenza-related complications in this immunologically-susceptible group.

However, it has been shown that influenza-specific IgG responses, as measured by antibody levels, are not impaired in HIV-infected individuals following vaccination, irrespective of CD4 count. In contrast, other studies in the subsequent years, using hemagglutination-inhibition assay, have shown that following vaccination with monovalent 2009 pandemic influenza A (H1N1) vaccine; HIV-infected adults generated significantly poorer antibody responses compared with HIV-uninfected persons [27], and that, HIV-infected adults did not generate durable seroprotective antibody response [28]. Future studies should investigate the association between reduced naturally-acquired influenza-specific CD4^+^ T-cell immunity and naturally-acquired influenza-specific antibody responses in HIV-infected individuals.

The main limitation of our study is that we did not investigate the effect of HIV and HAART on anti-influenza hemagglutinin (HA) antibodies. However, recently, a study by Wilkinson *et al.* has shown that in humans pre-existing influenza-specific CD4^+^ T-cells and not influenza-specific CD8^+^ T cells were associated with lower virus shedding and less severe illness in absence of detectable anti-influenza antibodies [29]. This above-mentioned study highlights the importance of CD4^+^ T-cell immunity in defence against influenza infection and introduces possibilities for development of influenza vaccines that will be able to target CD4^+^ T-cells, which will then potentially provide cross-protection against different influenza strains. Our study, therefore provides some evidence to suggest that the impaired influenza-specific CD4^+^ T-cell immunity may be responsible in part for the increased risk in influenza-associated complications in HIV-infected persons.

In conclusion, healthy African adults have a robust influenza-specific CD4^+^ T-cell immunity similar with other populations with a known high prevalence rate of influenza infection. This immunity is lost in HIV-infected adults and may increase the risk of influenza-related complications. Although initiation of HAART managed to suppress viral load and boost the CD4 count, it did not restore the influenza-specific immunity even after 12months on HAART, suggesting that this intervention alone may not rapidly reduce the risk of influenza-related complications. These data highlight the need to intensify influenza surveillance to determine the burden of disease in immunologically-susceptible African populations.

## Supporting Information

Figure S1 **Similar CD4^+^ T-cell proliferative responses to a polyclonal stimulus in HIV-infected adults compared to HIV-uninfected individuals.** (A) Peripheral blood mononuclear cells were stained with CFSE, stimulated with PHA for 7 days and proliferative responses were measured using flow cytometry. Black horizontal bars represent median and IQRs after background responses were subtracted. Statistical significance was analysed by the Mann-Whitney U test (CD≤350, n = 8; CD>350, n = 7; HIV−, n = 10).
(TIF)
