# Supplementary figures and images for: Naturally-Acquired Influenza-Specific CD4+ T-Cell Proliferative Responses Are Impaired in HIV-Infected African Adults
Source: PLoS One. 2012 Jun 8;7(6):e38628. doi: 10.1371/journal.pone.0038628 (PMC3371025; doi:10.1371/journal.pone.0038628)

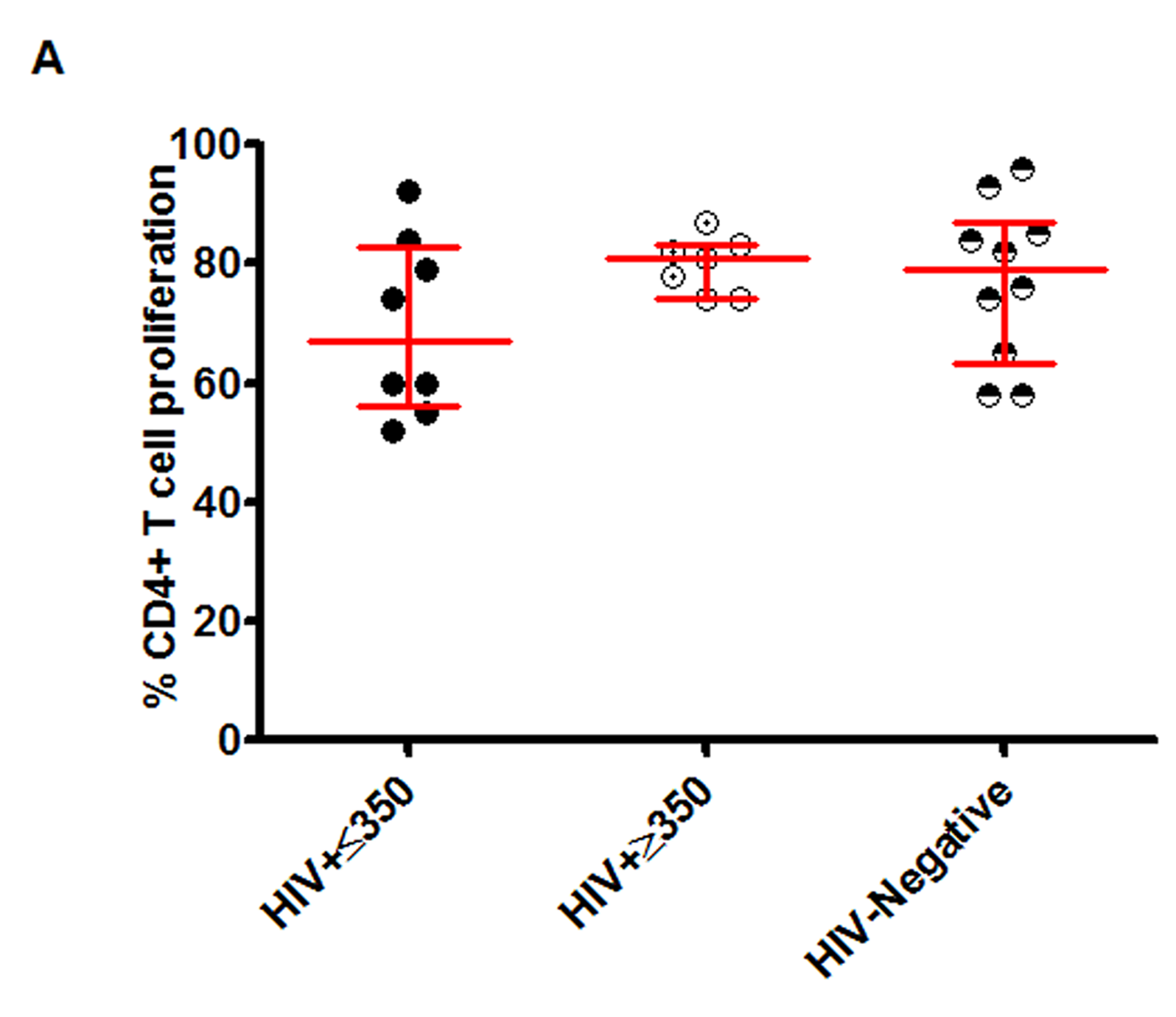

Supplement: Figure S1 — Similar CD4+ T-cell proliferative responses to a polyclonal stimulus in HIV-infected adults compared to HIV-uninfected individuals. (A) Peripheral blood mononuclear cells were stained with CFSE, stimulated with PHA for 7 days and proliferative responses were measured using flow cytometry. Black horizontal bars represent median and IQRs after background responses were subtracted. Statistical significance was analysed by the Mann-Whitney U test (CD≤350, n = 8; CD>350, n = 7; HIV−, n = 10). (TIF) [file pone.0038628.s001.tif]
